# Editorials

**Published:** 1890-08

**Authors:** 


### HOMŒOPATHIC PHYSICIAN,

A MONTHLY JOURNAL OF

HOMŒOPATHIC MATERIA MEDICA AND CLINICAL MEDICINE.

If our school ever give up the strict inductive method of Hahnemann, we are lost, and deserve only to be mentioned as a caricature in the history of medicine."—CONSTANTINE HERING.

Vol. X. AUGUST, 1890. No. 8.

### EDITORIALS.

THE INTERNATIONAL HAHNEMANNIAN ASSOCIATION'S LAST MEETING—an account of which will be found on other pages —proved to be the most successful, both in attendance and interest, ever held.

Conversation with the members from the various parts of the United States and Canada elicited the fact that the principles advocated by the I. H. A. are advancing, and that mongrelism is preparing to take a back seat.

An encouraging fact was the presence at the meeting of a number of young men who went for the purpose of learning. They did not go in vain, for they heard of nothing but Hahnemannian Homœopathy, and several expressed themselves to us as having received much profit.

Each year sees an addition to the membership, and it will not be long before the I. H. A. will be acknowledged throughout the world as the most progressive association of medical men ever organized. G. H. C.

---

ANTISEPTICS.—At last the ideal antiseptic and pus-destroyer has been found! We have the word of a man who has used it in a dozen cases with such excellent (?) results that he has rushed into print with a history of them. Some are completely cured of old and intractable troubles; others have mended, but are going rapidly "*toward*" cure.

Pyoktanin is its name, Germany is its nation, but New York beats the rest of the States in first proclaiming its station. There is color for all this; Pyoktanin is the well-known methylanilin, so much used by microscopists as coloring material when studying bacteria.

All hail! Pyoktanin—until it is found of no value, and then we shall hail something else. G. H. C.

